# Microglia and microbiome in schizophrenia: can immunomodulation improve symptoms?

**DOI:** 10.1007/s00702-023-02605-w

**Published:** 2023-02-21

**Authors:** Georg Juckel, Nadja Freund

**Affiliations:** Department of Psychiatry, Ruhr-University Bochum, LWL-University Hospital, Alexandrinenstr.1, 44791 Bochum, Germany

**Keywords:** Microglia, Microbiome, Schizophrenia, Immunomodulation

## Abstract

In this overview, influences of microglia activation and disturbances of the microbiome in the devastating disorder schizophrenia are discussed. Despite previous assumptions of a primary neurodegenerative character of this disorder, current research underlines the important autoimmunological and inflammatory processes here. Early disturbances of microglial cells as well as cytokines could lead to weakness of the immunological system in the prodromal phase and then fully manifest in patients with schizophrenia. Measurements of microbiome features might allow identifying the prodromal phase. In conclusion, such thinking would imply several new therapeutic options regulating immune processes by old or new anti-inflammatory agents in patients.

## Introduction

Schizophrenia is a mental illness that affects at least 24 million people worldwide (Tandon et al. 2009; WHO). The exact causes of schizophrenia are not known, but various factors, such as a genetic predisposition, imbalance of metabolic processes in the brain (dopamine and glutamate), stress, psychosocial influences, birth complications, and toxic and hormonal factors, are thought to play a role in the disease. In addition, (pre-)/perinatal infections are an important factor in the subsequent development of schizophrenia in the offspring (Brown and Derkits 2010). Here, epidemiological evidence has been reported in the literature linking maternal and fetal expression of inflammatory markers to the later development of schizophrenia (Brown et al. 2004; Buka et al. 2001; Nielsen et al. 2014), whereas the additional influence of pubertal stress associated with early childhood inflammation has to be taken into account (e.g., Giovanoli et al. 2013). However, because the identity of the pathogen (virus, bacterium, parasite) seems irrelevant (Brown et al. 2000; Brown and Susser 2002; Pearce 2001), it has been suggested that the effect of the maternal immune response on the fetal brain increases the risk for the child to later develop the disorder. Direct support for this hypothesis is mainly derived from animal studies of maternal immune activation (Meyer and Feldon 2012; Shi et al. 2003).

### Animal models of maternal immune activation

Activation of the maternal immune system during pregnancy in rodents has been conducted with several substances (for review see: Meyer 2014). One of the most common models of maternal immune activation (MIA) used to study schizophrenia is the polyinosinic:polycytidylic acid (polyI:C) animal model. PolyI:C is a synthetic, double-stranded RNA, as present in some viruses. Pregnant dams are treated with polyI:C on day 9 of pregnancy to induce a maternal immune response. Schizophrenia-relevant behavioral changes in the offspring have already been described repeatedly in this established model (Eßlinger et al. 2016; Fortier et al. 2004; Shi et al. 2005). Interestingly, similar schizophrenic behavioral changes have also been described in animals infected with a human influenza virus (Shi et al. 2003). As an established and already well-studied model system, PolyI:C is used to simulate viral infections. Thus, it meets predictive validity for schizophrenia-like pathology. PolyI:C injection elicits the same maternal immune response as infection with, for example, influenza, but the clinical picture is absent in the mother. The brain develops into a schizophrenia-like pathological state as a result of MIA (Meyer and Feldon 2010). Differences in, for example, receptor expression following MIA during development are already known to be marked in the early postnatal days and remain present throughout development (Garay et al. 2013; Mundorf et al. 2021). This implies that early neural changes may prime the brain for a schizophrenia-like state. On the other hand, it also implies that early manipulation of these neuronal changes by, for example, psychosocial interventions or medication administration could prevent the manifestation of schizophrenia. To date, early detection of symptoms of schizophrenia has been difficult. However, early detection of schizophrenia is known to be associated with a better course and less pronounced symptomatology (Häfner and Maurer 2006; Ruhrmann et al. 2010). Therefore, developmental impairments of offspring after polyI:C infection during pregnancy as well as resulting neurobiological changes during the course might be helpful to develop markers for valid early diagnosis.

### Activated microglia in schizophrenia

**Fig. 1:**
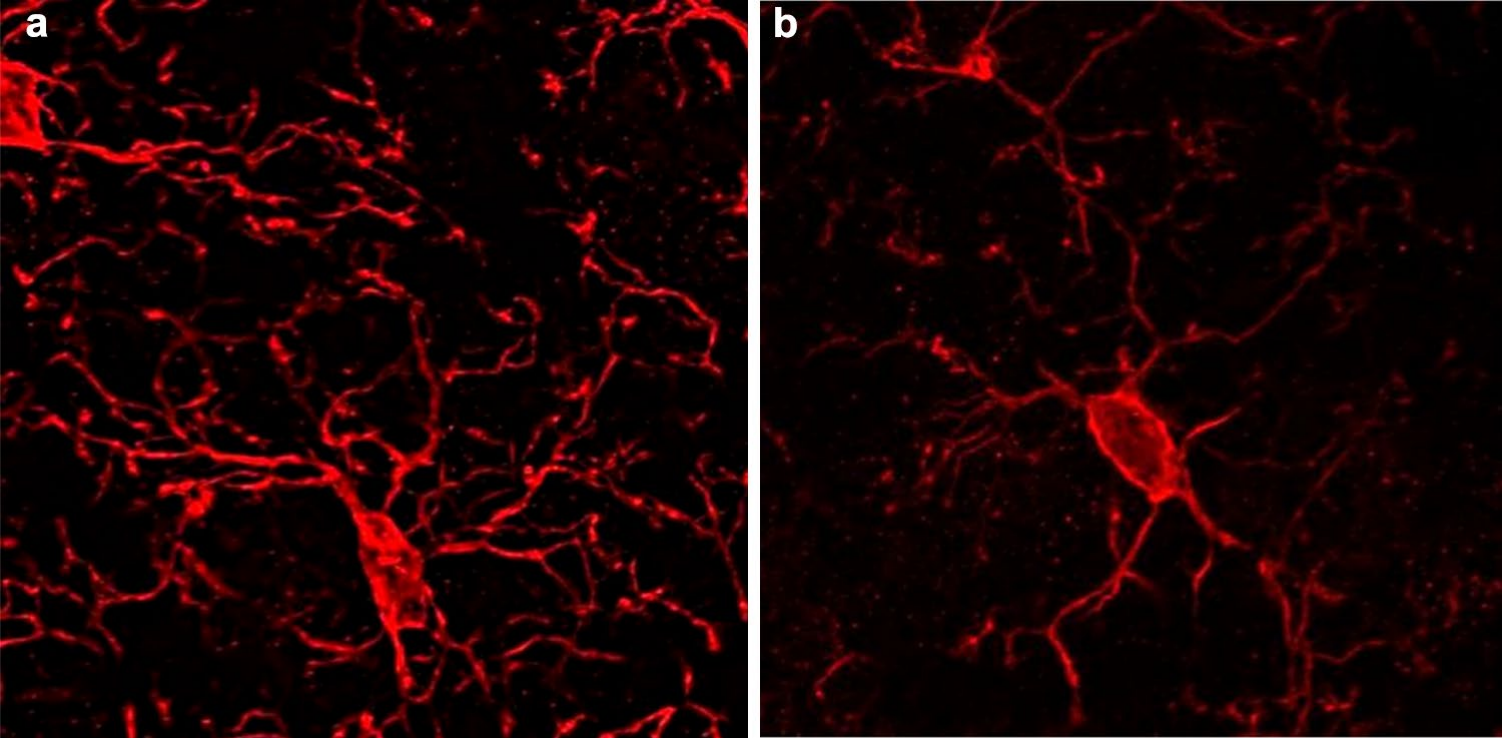
Confocal microscopy of resting (**a**) and activated (**b**) microglia cells

Microglia represent the immune cells of the brain and mediate phagocytosis of diseased or damaged cells, particularly during pathological processes. The origin of microglia in the CNS is widely debated, with recent studies showing that microglia arise early during development from precursor cells in the embryonic yolk sac. Prior to the formation of the fetal circulation, these cells populate the early embryonic brain. In mice, the earliest detectable settlement of cells with myeloid functions (primitive microglia) in the brain occurs at time point E8.5/E9.0 (Alliot et al. 1991; Ginhoux et al. 2013). As development continues, microglial proliferation increases sharply until the second week after birth. Subsequently, the microglial population remains at constant levels in adulthood due to longevity and limited self-renewal, independent of circulating precursors such as macrophages. Microglia are distributed throughout the CNS, but there are region-specific differences in their density, molecular phenotype, morphology, and function (de Haas et al. 2008; Lenz et al. 2013; Schwarz et al. 2012). Under physiological conditions, microglia in the adult brain have long, thin, highly ramified extensions and "monitor" their microenvironment. These are so-called resting microglia. In case of inflammation or injury, the microglia change their morphology. The extensions become smaller, wider, and the cell bodies (somata) become thicker. These cells are defined as transitioning cells (see Fig. 1). As they progress, these activated microglia adopt an amoeboid morphology and migrate to the site of inflammation (Juckel et al. 2011). These processes have been previously described using in vivo "two-photon imaging" in the neocortex (Nimmerjahn et al. 2005). At the site of inflammation, activated microglia strongly engage in pro-inflammatory signaling pathways and secrete cytokines and chemokines to recruit additional innate immune cells (Kreutzberg 1996). The main function of microglia in this context is phagocytosis of apoptotic cells/cell debris to confine tissue damage (Hanisch and Kettenmann 2007). Furthermore, microglia secrete anti-inflammatory molecules and growth factors to limit chronic inflammation and promote repair (Kreutzberg 1996). Microglia can exhibit both classical pro-inflammatory activation (M1 microglia) and alternative anti-inflammatory activation (M2 microglia). These subpopulations of M1 and M2 microglia act in concert, but it remains unclear to what extent the different molecular phenotypes are responsible for resolving or exacerbating inflammation. In numerous neurodegenerative diseases, microglia are in a chronic pro-inflammatory state, and ongoing activation by neurotoxic processes, such as secretion of NO, antioxidants, etc., is thought to exacerbate disease progression. It is hypothesized that the transition from pro-inflammatory M1 to anti-inflammatory M2 microglia is inhibited by the microenvironment in the brain of schizophrenic patients. This imbalance of a) lack of M2 polarization and b) increased M1 activation gradually leads to impaired neuronal functions as the disease progresses (Meyer 2011; Nakagawa and Chiba 2014).

Activation of microglia leads to neuropil reduction and alteration of synaptic plasticity, which was repeatedly found in patients with schizophrenia and in animal models (Eßlinger et al. 2016; Juckel et al. 2011; Manitz et al. 2013, 2016). Hereby, among others the secretion of nitric oxide (NO) plays an important role (Esshili et al. 2020; Wegrzyn et al. 2021). Altogether, these findings confirm the well-known brain development hypothesis of schizophrenia (Weinberger 1987).

Based on the now well-established "gut-brain" axis, a pathophysiologically important interaction between the activity of central nervous microglia and the gut microbiome is hypothesized in schizophrenic disorders. First evidence confirming this hypothesis has already been secured in human and animal experiments (Cowan and Petri 2018; Juckel et al. 2021).

### Disrupted microbiota in schizophrenia

Over the past few years, immunopathogenesis has emerged as one of the most compelling etiopathological models of schizophrenia. A chronic, immune-based, low-grade inflammatory background is suggested in this devastating disorder (Khandaker et al. 2015). Increasing evidence points toward a prominent role of the adaptive immune system in schizophrenia, with potential alterations in defense mechanisms, such as altered T cell function and a shift toward B cell immunity (Debnath 2015). Immune cells have the ability to infiltrate the brain and mediate a neuroimmune cross-talk through activation of microglia (Juckel et al. 2011; Manitz et al. 2013) and production of pro-inflammatory cytokines and reactive oxygen species. These processes lead to neuroinflammation and mediate neuroprogressive and neurodegenerative changes in schizophrenia (Laskaris et al. 2016). Adaptive immunity is mainly driven by T cell and B cell populations but can also be influenced by the microbiome. The gastrointestinal microbiota is a complex ecosystem with great range of numbers and types of organismal diversities and refined genomic structure. Herby, alpha diversity means effective numbers of species (types of organisms) within one microbiome probe (one given body habitat described by richness and evenness), and beta diversity measures the relationship between the numbers of species to the species' abundance within a sample of all probes. Microorganisms of the intestinal flora support the immune system and are in constant exchange with the brain via the gut–brain axis. A direct connection between the intestine and the brain exists via the vagus nerve enables an exchange of certain messenger substances. Recent studies suggest a direct impact of the gut flora, the microbiome on mental illnesses such as schizophrenia. This impact is supported by current studies that have found decreased microbiome diversity in patients with schizophrenia (Zheng et al. 2019). Microbiome diversity in patients with schizophrenia is in part associated with a specific schizophrenic phenotype, symptom severity, cognitive deficits, and treatment response (Bioque et al. 2021; Dickerson et al. 2017; Nguyen et al. 2018). Several lines of evidence suggest that dysbiosis fits very well with known hypotheses of schizophrenia pathogenesis, especially those focusing on inflammation and especially neuroplasticity (Szeligowski et al. 2020).

Early stress is an established risk for the development of psychiatric disorders, e.g., failed metabolic programming of the fetus leads to schizophrenia in individuals previously exposed to prenatal stress. Here, the interaction between hereditary factors and the intrauterine environment accelerates to the onset of the disease by disrupting the course of normal brain development (Garcia-Rizo and Bitanihirwe 2020).

Studies have shown that early life stress in form of postweaning social isolation in rats can lead to long-lasting alterations in the gut microbiota, a possibility that would contribute to the development of abnormal neuronal and endocrine functions and behaviors. These abnormalities may play a central role in schizophrenia (Dunphy-Doherty et al. 2018). Other studies have used animals with a focus on transplanting fecal microbiota from, for example, patients with schizophrenia into specific pathogen-free mice. These experiments have examined whether this transplantation effect causes schizophrenia-like behavioral abnormalities, such as psychomotor hyperactivity and impaired learning and memory, as well as alterations in kynurenine, dopamine, and serotonin pathways in recipient animals (Zhu et al. 2020). Others have found that the metabolic phenotypes of the cortex, cerebellum, and striatum are substantially different in recipient mice of schizophrenia microbiota. These data suggest that alterations in glycerophospholipid and fatty acid metabolism are associated with the occurrence of schizophrenia-related behaviors (Liang et al. 2019). The polyI:C mouse model also showed changes in the microbiome compared to control animals (Juckel et al. 2021). During development at postnatal day 30, the abundance of certain microbiota families differs between the model and controls in a sex-specific manner. Very interestingly, this affect cannot be found in adult animals. Supporting these data, altered microbiota diversity and an inflammatory response of the gut were also reported in a poly I:C rat model (Li et al. 2021).

### Substance-induced modifications of microbiome and microglia

While there is evidence of negative effects of antibiotic treatments in patients with schizophrenia (Klein-Petersen et al. 2021; Minichino et al. 2021), new drug therapy approaches targeting inflammation and the microbiome show promising effects on the so-called positive and negative symptoms of schizophrenia (Fitton et al. 2022). Minocycline, an antibiotic that is completely absorbed by the small intestine and crosses the blood–brain barrier, indicated a beneficial effect on negative symptoms in some clinical studies but results are mixed (Kishimoto et al. 2018). Similar reports come from the use of the anti-inflammatory drug acetylic acid (aspirin) as add-on therapy. While some studies claim a beneficial effect (Laan et al. 2010; Attari et al. 2017), others find no differences compared to placebo (Weiser et al. 2021). In contrast, meta-analysis reveals that the anti-inflammation drug celecoxib holds a positive effect when used as adjunctive (Müller et al. 2002; Zheng et al. 2017). Specifically targeting the gut microbiome, one study administered a combination of vitamin D and probiotics to schizophrenia patients and succeeded in reducing symptoms (Ghaderi et al. 2019). However, overall the results for the administration of pre- and probiotics are mixed (Szeligowski et al. 2020).

Taken together, the neuronal and immunological effects of these drugs in the context of schizophrenia are insufficiently understood. Microglial activity seems to play a key role, presumably regulated by the gut microbiome, but also by enteric neurons and immunocompetent cells (e.g., macrophages) (Boehme et al. 2020). The use of rodent models for schizophrenia might help understanding the underlying mechanisms and identifying suitable treatment options. Several studies have shown that treatment with the antibiotic minocycline can counteract the effect of prenatal exposure to poly I:C (Alari-Pahissa et al. 2016; Mattei et al. 2014, 2017; Shemer et al. 2021; Xia et al. 2020; Zhu et al. 2014). Not only an improvement in behavior but also specifically a protective effect regarding microglia was shown. The expression of iNOS and the activation of microglia were reduced (Giovanoli et al. 2016; Zhu et al. 2014). Minocycline normalized the cytokine production of microglia (Mattei et al. 2014) and prevented changes in their gene expression of a wide variety of genes related to e.g., inflammation, cell migration, phagocytosis, and synaptic plasticity (Mattei et al. 2017). While the authors are not aware of any study on aspirin in the Poly I:C model, in vitro studies show that aspirin can reduce microglia’s production of the pro-inflammatory cytokine TNF-α (Pettit et al. 2013) and might inhibit poly I:C induced microglia activation (Wu et al. 2020). In a lipopolysaccharide-induced mouse model for autism (maternal immune activation appears later in gestation compared to the Poly I:C model for schizophrenia), a probiotic approach was able to ameliorate the effects of the maternal immune activation (Lin et al. 2022).

### Outlook

Changing the view in understanding schizophrenia as both a neurodegenerative and a neuro-inflammatory disease provides several interesting perspectives for future research. The interaction of brain and body, here the gut, plays an important role for the pathophysiological discussion also in this disorder. In this way, some of the somatic symptoms in the patients with schizophrenia resulting in a reduced life expectancy will get clearer in their biological underlying factors. Thus, concerning this, but also other features of this serious disease, new therapeutic possibilities occur in this field increasing our strategies to improve patients’ state and trait characteristics.

## Data Availability

Data available on request.
